# Use of Secukinumab in a Cohort of Erythrodermic Psoriatic Patients: A Pilot Study

**DOI:** 10.3390/jcm8060770

**Published:** 2019-05-31

**Authors:** Giovanni Damiani, Alessia Pacifico, Filomena Russo, Paolo Daniele Maria Pigatto, Nicola Luigi Bragazzi, Claudio Bonifati, Aldo Morrone, Abdulla Watad, Mohammad Adawi

**Affiliations:** 1 Department of Dermatology, Case Western Reserve University, Cleveland, OH 44124, USA; 2 Clinical Dermatology, IRCCS Istituto Ortopedico Galeazzi, 20161 Milan, Italy; paolo.pigatto@unimi.it; 3 Department of Biomedical, Surgical and Dental Sciences, University of Milan, 20122 Milan, Italy; 4 Young Dermatologists Italian Network (YDIN), Centro Studi GISED, 24121 Bergamo, Italy; 5 San Gallicano Dermatological Institute, IRCCS, 00144 Rome, Italy; alessia.pacifico@gmail.com (A.P.); claudio.bonifati@ifo.gov.it (C.B.); aldomorrone54@gmail.com (A.M.); 6 Dermatology Section, Department of Clinical Medicine and Immunological Science, University of Siena, 53100 Siena, Italy; file.russo@libero.it; 7 School of Public Health, Department of Health Sciences (DISSAL), University of Genoa, 16132 Genoa, Italy; robertobragazzi@gmail.com; 8 NIHR Leeds Biomedical Research Centre, Leeds Teaching Hospitals NHS Trust, Leeds Institute of Rheumatic and Musculoskeletal Medicine, University of Leeds, Leeds LS7 4SA, UK; watad.abdulla@gmail.com; 9 Department of Medicine ‘B’, Sheba Medical Center, Tel-Hashomer and Sackler Faculty of Medicine, Tel Aviv University, 5265601 Tel Aviv, Israel; 10 Padeh and Ziv Hospitals, Azrieli Faculty of Medicine, Bar-Ilan University, 5290002 Ramat Gan, Israel; adawimo1802@gmail.com

**Keywords:** erythrodermic psoriasis, secukinumab, addiction, smoking, alcohol, cannabis

## Abstract

Erythrodermic psoriasis (EP) is a dermatological emergency and its treatment with secukinumab is still controversial. Furthermore, no data exist regarding the prognostic value of drug abuse in such a condition. We performed a multi-center, international, retrospective study, enrolling a sample of EP patients (body surface area > 90%) who were treated with secukinumab (300 mg) during the study period from December 2015 to December 2018. Demographics and clinical data were collected. Drug abuses were screened and, specifically, smoking status (packages/year), cannabis use (application/week) and alcoholism—tested with the Alcohol Use Disorders Identification Test (AUDIT)—were assessed. All patients were followed for up to 52 weeks. We enrolled 13 EP patients, nine males, and four females, with a median age of 40 (28–52) years. Patients naïve to biologic therapy were 3/13. Regarding drug use, seven patients had a medium-high risk of alcohol addiction, three used cannabis weekly, and seven were smokers with a pack/year index of 295 (190–365). The response rate to secukinumab was 10/13 patients with a median time to clearance of three weeks (1.5–3). No recurrences were registered in the 52-week follow-up and a Psoriasis Area Severity Index (PASI) score of 90 was achieved. The entire cohort of non-responders (*n* = 3) consumed at least two drugs of abuse (alcohol, smoking or cannabis). Non-responders were switched to ustekinumab and obtained a PASI 100 in 24 weeks. However, given our observed number of patients using various drugs in combination with secukinumab in EP, further studies are needed to ascertain drug abuse prevalence in a larger EP cohort. Secukinumab remains a valid, effective and safe therapeutic option for EP.

## 1. Introduction

Erythroderma is an uncommon and severe dermatological manifestation of a variety of diseases. The most common form of erythroderma is erythrodermic psoriasis (EP), which accounts for 1–2.25% of all psoriatic patients, with a male predominance as demonstrated by a male to female ratio of 3:1 [1]. EP clinically manifests with diffuse erythema (body surface area (BSA) > 75%) involving also skin folds with or without exfoliate dermatitis.

Several triggers have been described to elicit EP in predisposed subjects such as environmental factors (sunburn, alcoholism, and infections), drugs (lithium, anti-malarial drugs), and the rebound phenomenon following discontinuation of anti-psoriatic treatments (oral steroids or methotrexate) [1]. However, the pathogenesis of EP remains elusive, which can limit a physician’s capability to deliver safe and effective therapy. In 2010, the National Psoriasis Foundation (NPF) published a guideline describing the current evidence regarding EP treatment, stating that cyclosporine and infliximab should be the first line treatment in acute and unstable patients, whilst methotrexate and acitretin are recommended in more stable patients [2].

Despite this clear advice, prominent limitations included that few high-quality studies assessing EP treatment were present in the literature [2]. In a clinical setting, EP treatment faces two other prominent challenges, namely the difficulty in differential diagnosis and in implementing a biological approach that rules out non-inflammatory conditions. Although histological confirmation is mandatory in suspected EP cases, it is sometimes challenging due to the potential lack of histological parameters resembling classical psoriasis, such as parakeratosis or acanthosis [1]. 

The exclusion of neoplastic causes (Sézary syndrome) is mandatory if biologics are the selected approach. In fact, in the last 5 years, neoplasia has been a relative contraindication [3]. The NPF guidelines did not include IL-17 inhibitors [2], such as secukinumab, and recently two case series studies described the use of secukinumab in EP patients [4,5]. Current evidence seems to support the use of secukinumab in EP patients, even though there is a dearth of data concerning potential predictors of responsiveness in these patients. 

Remarkably, among psoriatic patients, alcohol use/abuse and smoking are described and linked to both psoriasis development and exacerbations but are not studied in EP [6,7,8,9,10]. Conversely, the prevalence of cannabis users among psoriatic patients and the effect of cannabis use on psoriasis are still missing. Furthermore, in vitro or murine studies explored keratinocyte changes only in response to a single cannabis compound [11]. Thus, due to the increasing prevalence of cannabis users in the general population and also its promoting role in medicine [11], reports focusing on the effect in psoriasis are needed.

The current study aimed to evaluate (i) first the efficacy and safety of secukinumab in psoriatic erythroderma and (ii) second to describe the prevalence of drug abuses, namely alcohol, tobacco, and cannabis smoking, in EP patients.

## 2. Experimental Section

This multi-center, international, retrospective, pilot study enrolled a sample of EP patients (BSA > 90%) treated with a loading dose of 300 mg subcutaneous secukinumab at weeks 0, 1, 2, 3 and 4, followed by 300 mg every 4 weeks, in the period from December 2015 to December 2018.

All erythrodermic patients were biopsied and malignancies were ruled out by complete blood count, blood smear, transaminases, lactate dehydrogenase (LDH), gamma-glutamyl transferase (GGT), anion gap, Sézary cell search, and total body computed tomography. Smoking history (pack/years), cannabis use (smoking episodes/week) and alcohol use (Alcohol Use Disorders Identification Test (AUDIT)) status were assessed.

AUDIT is a 10-question screening tool (0–40 points) developed by the World Health Organization (WHO) in order to evaluate alcohol consumption, drinking behavior, and alcohol-related complications. According to AUDIT, patients are stratified as follows: 0–7 points indicate a low risk, 8–15 points a medium risk, 16–19 points a high risk, and 20–40 points a probable addiction.

All EP patients underwent a 52-week follow-up to evaluate recurrent erythrodermic episodes.

Demographics and clinical charts were recorded, including: age; gender; previous Psoriasis Area Severity Index (PASI) score before erythroderma, if any; previous anti-psoriatic therapy; biologic therapy exposure; secukinumab response; side effects; drug use history; PASI and Dermatologic life quality index (DLQI) at weeks 8, 12, 16, 24, and 52. We stratified erythroderma clearance (BSA < 75%) based on PASI 75, PASI 90, PASI 100.

## 3. Results

### 3.1. Study Population

In the current study, 13 EP patients (female/male ratio equal to 9/4), with a median age of 40 (28–52), and body mass index of 24 (22–27) kg/m^2^ were included. Family history of psoriasis was positive in 9/13 patients.

### 3.2. Drug History

In Table 1 we assessed drug history. Only 3/13 patients were naïve to biologic therapy. Among patients treated with biologics, eight had switched more than two biologics. Furthermore, 8/13 had a previous episode of erythroderma and six patients had more than two episodes. Drug history indicated that some of the EP patients had previously received therapeutic agents that could potentially trigger psoriasis, namely four underwent beta blockers, three received angiotensin II blockers (ARBs), two patients received angiotensin-converting enzyme (ACE) inhibitors, and one patient was previously treated with thiazide diuretics.

### 3.3. Drug Abuses and Comorbidities

Drug abuse screening revealed that seven patients had a medium-high risk of alcohol abuse, three patients used cannabis on a weekly basis, and seven patients were smokers with a pack/year index of 295 (190–365). The comorbidities represented in our cohort included: dyslipidemia (five patients), hypertension (three patients), osteoporosis (two patients), atrial fibrillation (one patient) and pulmonary tuberculosis (one patient), respectively (Table 2).

### 3.4. Clinical Response to Secukinumab

**Figure 1:**
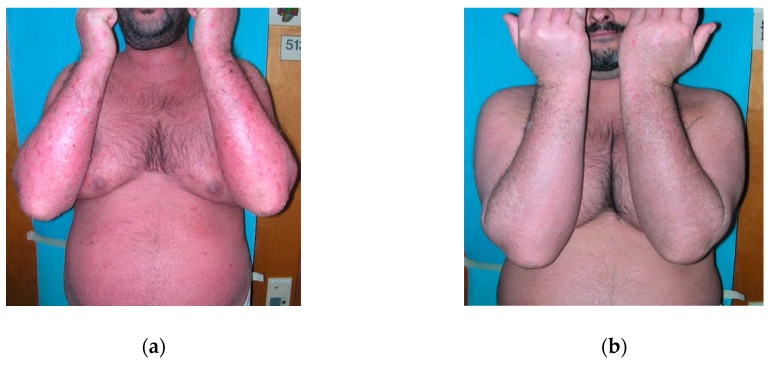
A 34-year-old patient with erythrodermic psoriasis that underwent secukinumab therapy. (**a**) Erythrodermic patient before treatment, (**b**) Patient after three weeks of secukinumab treatment.

Clinical and therapeutic data are summarized in Table 3. The median value of the last recorded PASI was 10 (7–15). Responders to secukinumab were 10/13 (Figure 1a,b) and the median clearing time was three (1.5–3) weeks.

**Figure 2:**
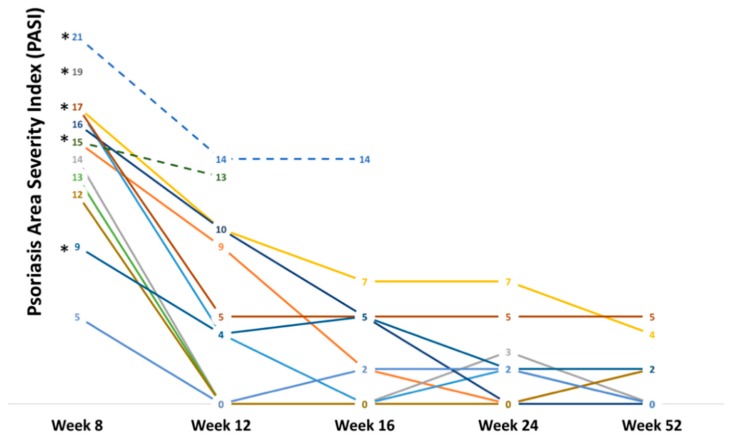
PASI trends in erythrodermic patients from week eight to week 52. * Patients that displayed more than one type of drug use (tobacco, cannabis, alcohol).

Side effects were reported in 5/13 patients and remarkably were the only cause of treatment interruption, in contrast to other previously reported cases series [4,5]. All patients were on continuous secukinumab treatment and no recurrences were registered in the 52 weeks of follow up. After recovering from erythroderma at week eight, four patients achieved PASI 75, while none achieved PASI 90 or PASI 100. At week 52, five patients achieved PASI 90 and five achieved PASI 100. Interestingly, looking at the PASI trends of this cohort (Figure 2), all three non-responders used two out of three of the aforementioned drugs (alcohol, cannabis, and smoking) and no recorded comorbidities. Non-responders were switched to ustekinumab (90 mg) and obtained a PASI 100 in 24 weeks.

## 4. Discussion

Our study further supports that secukinumab is an effective therapy in EP and suggests that the use of recreational and accepted drugs (alcohol, cannabis, and tobacco) is prevalent in EP patients.

Furthermore, in the literature, EP patients treated with secukinumab had 16 [4] or 24 [5] months of follow up, lacking an assessment of long-term DLQI. Thus we assessed DLQI at 8, 12, 16, 24, and 52 weeks and found that secukinumab contributed to the improvement, not only in skin disease, but also in the long-term quality of life of EP patients. In our cohort, EP patients that responded to secukinumab did not exhibit recurrences and maintained long-term responsiveness to the drug. This study further supports the results described in a retrospective 52-week, observational, multicenter study, evaluated by Galluzzo et al., which suggested long-term efficacy of secukinumab in plaque psoriasis [12].

Focusing on EP patients, we assessed for the first time in detail the timing related to clearance of erythroderma, and after that, how secukinumab managed to clear the residual plaque psoriasis during the 52-week follow-up period. These two parameters together, are of pivotal importance in the clinical setting to guide therapeutic decisions made by dermatologists. In addition, the 52-week follow-up data highlighted that the EP responders to secukinumab achieved at least PASI 90 after clearing EP.

Among the three patients who did not respond to secukinumab therapy, one patient developed generalized urticaria at week three, the second patient experienced recurrent oral candidiasis and stopped the drug at week 12, and the third patient lost response at week 16. Remarkably, the second non-responder also smoked cannabis. Non-responders have not previously been treated with ustekinumab, and in accord with the recent real-life data on secukinumab non-responders, they were switched to ustekinumab and achieved a complete remission [13]. Ustekinumab is an IL-12/IL-23 blocker that targets the p40 subunit shared by these two cytokines. Furthermore, IL-12 plays a pivotal role in T helper cell type 1 (Th-1) polarization, as does IL-23 in Th-17 polarization [14]. We interpret the non-responsiveness of our patients as potentially due to the development of anti-secukinumab antibodies or up-regulation of Th-1-related pro-inflammatory cytokines, as previously demonstrated by Zaba et al. [15].

Evaluation of clinical characteristics in secukinumab non-responders indicated that all three had a familial history of EP and had used more than one drug, including smoking, alcohol, and cannabis. However, none of them were treated with any drug known to trigger psoriasis.

In the literature, the prevalence of drug abuse in the rare subset of EP is not reported, conversely, in plaque psoriasis patients several authors addressed the problem of drug abuse prevalence (alcohol and tobacco smoking) and its impact on anti-psoriatic therapies [7,8,16,17].

Alcohol intake and, consequently, also the abuse, may favor psoriasis-related systemic inflammation by promoting lipopolysaccharide (LPS) translocation from intestine to blood flow, increasing the pro-inflammatory activation of several immune cells, including lymphocytes (producing TNF-α and IFN-γ) and monocytes/macrophages (producing TNF-α), and directly by triggering keratinocytes pro-inflammatory activation via keratinocyte growth factor receptor (KGFR) [9]. These observations are further supported by Brenaut and colleagues, who conducted a meta-analysis on the epidemiological link between psoriasis and alcohol intake, and found that alcohol is a risk factor in developing psoriasis [17]. Furthermore, Qureshi et al. described a correlation between heavy beer intake and psoriasis severity during exacerbation [8]. This concept is translatable to EP patients, where erythroderma is an acute and very severe exacerbation of pre-existent psoriasis. Thus, alcohol abuse seems to increase TNF-α levels and may theoretically explain a possible lack or loss of response to IL-17 blockers, as with secukinumab in our EP patients.

Tobacco smoking and its link with psoriasis was assessed by Armstrong and colleagues in a large meta-analysis, involving 28 studies. They found an odds-ratio (OR) of 1.78 (95% confidence interval = 1.52–2.06) and a higher PASI in psoriatic smokers compared to non-smokers [16]. Remarkably, psoriasis severity gradually increases with the number of cigarettes smoked per day [17], but may benefit from a stop in smoking [18,19]. The nicotine contained in cigarettes activates nicotinic acetylcholine receptors (nAChRs) on the surface of dendritic cells, macrophages, endotheliocytes and keratinocytes, leading to an increased Th-1/Th-17 polarization of naïve T cells and to an increased production of pro-inflammatory cytokines, such as TNF-α, IL-12, IL-17, IL-23, IL-1β and IFN-γ [20]. These are all capable of decreasing the therapeutic effects of both TNF-α [7] and IL-17 blockers.

Conversely, fragmentary data exist regarding the effects of cannabis on the immune system and skin [21,22], but no data have been published about cannabis smoking in psoriatic patients or in murine models of psoriasis. However, some purified extracts derived from cannabis may inhibit in vitro keratinocyte proliferation [21] and Th-17 cell-related cytokine production in a dose-dependent manner [22]. Cannabinoids mainly interact with two receptors, cannabinoid-1 receptor (CB1R) and (CB2R), and both inhibit adenylate cyclase and activate mitogen-activated protein kinase (MAPK) [11]. This theoretically contrasts the anti-psoriatic function of apremilast, with regard to the intracellular cyclic adenosine monophosphate (AMPc) increase due to phosphodiesterase-4 inhibition. CB1R is prevalently present in keratinocytes, whilst CB2R is prevalent in immune cells, such as T cells and monocytes/macrophages [11]. Upon stimulation in the presence of purified cannabis extracts, namely cannabidiol (CBD) and tetrahydrocannabinol (THC), Th-17 cells massively decrease both transcription and release of IL-17A [22], which may theoretically act synergistically with IL-17 blockers. This aspect may be also confirmed by reports that list candidiasis as a side effect of both IL-17 blockers and chronic cannabis use [23]. Consequently, patients under IL-17 blockers that use cannabis may be exposed to a higher risk of candidiasis. Remarkably, Russo and colleagues pointed out that, in order to evaluate the global effect of cannabis, it is necessary to take into consideration the synergism existing among different cannabis compounds that altogether determine the final so-called entourage effect, capable of enhancing or even obscuring the properties of single compound [24]. Furthermore, no studies evaluated how smoking cannabis can modify these compounds and their biological effect. Thus, this is the first report to evaluate this relevant use of such drugs in a cohort of patients affected by EP, a chronic systemic inflammatory disease.

Moreover, both smoking and alcohol consumption were found to increase IL-17 and TNF-α production [9,12,16], corroborating our hypothesis that drug use may promote systemic inflammation, contributing to less favorable results from anti-psoriatic therapies.

The main limitation of the present study remains the small sample of enrolled patients, which was due to EP rarity and due to the fact secukinumab is still off-label in treating EP. Therefore, we cannot conclude that drug use in the non-responding patient group was causal. Other plausible reasons for the failed response in the small number of patients with addiction problems in the present cohort might well be insufficient compliance, even though all of our patients regularly attended dermatological appointments and reported to have auto-injected secukinumab.

## 5. Conclusions

Although not conclusive, our preliminary results in EP patients treated with secukinumab enlighten two presently unmet needs: (i) the need of therapy-specific biomarkers/prognostic factors and (ii) the prevalence of drug use in EP.

In conclusion, secukinumab may be a safe and effective treatment in EP, however, larger studies are needed to validate our results.

## Figures and Tables

**Table 1:** Pharmacological history in our cohort.

Variables	EP (*n* = 13)
Last anti-psoriatic therapy (*N* (%))	
Methotrexate	1 (7.7)
Phototherapy	1 (7.7)
Adalimumab	4 (30.8)
Etanercept	2 (15.4)
Ustekinumab	2 (15.4)
Apremilast	1 (7.7)
Combination therapy (MTX + Etanercept)	3 (23.1)
Biologics naïve (*N* (%))	3 (23.1)
Biologics switching (*N* (%))	10 (76.9)
1	2 (20.0)
2	5 (50.0)
3	1 (10.0)
>3	2 (20.0)
Other drugs capable to aggravate psoriasis (*N* (%))	
Beta-blockers	4 (30.8)
ACE inhibitors	2 (15.4)
ARBs	3 (23.1)
Thiazides diuretics	1 (7.7)

ACE: Angiotensin-converting enzyme, ARBs: Angiotensin II receptor blockers, EP: erythrodermic psoriasis, MTX: Methotrexate.

**Table 2:** Prevalence of drug abuses in our cohort.

Addictions	EP (*n* = 13)
Smokers (*N* (%))	7 (53.8)
Pack/year (median (IQR))	295 (190–365)
AUDIT test (median ± SD)	9 (4–14)
Zone I (0–7 points) (*N* (%))	6 (46.2)
Zone II (8–15 points) (*N* (%))	6 (46.2)
Zone III (16–19 points) (*N* (%))	1 (7.7)
Zone IV (20–40 points) (*N* (%))	0 (0.0)
Cannabis use (*N* (%))	3 (23.1)

AUDIT: Alcohol Use Disorders Identification Test, EP: erythrodermic psoriasis, IQR: Interquartile range, SD: standard deviation.

**Table 3:** Clinical and therapeutic records in our cohort.

Variables	EP (*n* = 13)
Last control PASI (median (IQR))	10 (7–15)
Secukinumab responders (*N* (%))	10 (76.9)
Secukinumab non-responders (*N* (%))	3 (23.1)
Previous erythroderma episodes (*N* (%))	8 (61.5)
1	2 (25.0)
2	3 (37.5)
3	1 (12.5)
>3	2 (25.0)
Erythroderma clearing time (median (IQR), weeks)	3 (1–5.3)
PASI (median (IQR))	
Week 8	15 (13–17)
PASI 75 (*N* (%))	4 (30.8)
PASI 90 (*N* (%))	0 (0.0)
PASI 100 (*N* (%))	0 (0.0)
Week 12	4.5 (0–10)
PASI 75 (*N* (%))	5 (38.5)
PASI 90 (*N* (%))	3 (23.1)
PASI 100 (*N* (%))	4 (30.8)
Week 16	2 (0–5)
PASI 75 (*N* (%))	1 (7.7)
PASI 90 (*N* (%))	5 (38.5)
PASI 100 (*N* (%))	4 (30.8)
Week 24	2 (0–2.75)
PASI 75 (*N* (%))	1 (7.7)
PASI 90 (*N* (%))	5 (38.5)
PASI 100 (*N* (%))	4 (30.8)
DLQI (median (IQR))	
Week 8	17 (13–22)
Week 12	12 (9–17)
Week 16	11 (7–16)
Week 24	8 (6–12)
Week 52	8 (5–12)
Side effects (*N* (%))	5 (38.5)
Recurrent oral candidiasis	1 (20.0)
Urticaria	1 (20.0)
Injection-site pain	3 (60.0)

DLQI: Dermatologic Life Quality Index, EP: erythrodermic psoriasis, IQR: Interquartile range, MTX: Methotrexate, PASI: Psoriasis Area Severity Index.
